# Synchronization of two cavity-coupled qubits measured by entanglement

**DOI:** 10.1038/s41598-020-69903-1

**Published:** 2020-07-31

**Authors:** Tian-tian Huan, Ri-gui Zhou, Hou Ian

**Affiliations:** 1 Institute of Applied Physics and Materials Engineering, University of Macau, Macau, China; 2 College of Information Engineering, Shanghai Maritime University, Shanghai, 201306 China; 3 Zhuhai UM Science & Technology Research Institute, Zhuhai, Guangdong China

**Keywords:** Qubits, Single photons and quantum effects, Quantum information

## Abstract

Some nonlinear radiations such as superfluorescence can be understood as cooperative effects between atoms. We regard cooperative radiations as a manifested effect secondary to the intrinsic synchronization among the two-level atoms and propose the entanglement measure, concurrence, as a time-resolved measure of synchronization. Modeled on two cavity-coupled qubits, the evolved concurrence monotonically increases to a saturated level. The finite duration required for the rising to saturation coincides with the time delay characteristic to the initiation of superfluorescence, showing the role of synchronization in establishing the cooperation among the qubits. We verify concurrence to be a good measure of synchronization by comparing it with asynchronicity computed from the difference between the density matrices of the qubits. We find that the feature of time delay agrees in both measures and is determined by the coupling regimes of the cavity-qubit interaction. Specifically, synchronization is impossible in the weak coupling regime.

## Introduction

Centuries ago, Huygens studied the correlation among the motions of pendulums and discovered the synchronization pattern of these individual oscillators under the influence of a common oscillator they are coupled to^1^. How synchronizations arise in different situations has since remained a problem of interest^2^. In recent years, the study of the classical phenomenon has been revived under the quantum regime. Synchronization is observed between a pair of nanomechanical oscillators^3^ and on the motions of the Cooper pairs among Josephson-insulated superconducting islands^4^. It is also ubiquitously predicted between a qubit and an oscillator^5^, between a cavity field and an oscillator^6^, between two oscillators^7^, among a trapped group of cold atoms^8^, and even between a quantum Van der Pol oscillator and an external drive^9^.

Here we study the synchronization of two qubits coupled indirectly to each other through a cavity field, in specific relevance to the quantum optical phenomenon of superfluorescence^10,11^. This nonlinear fluorescent effect embodies Dicke’s formulation of superradiance^12^, where the nonlinearity exemplifies in the $N^{2}$-dependence of the emitted radiation intensity from an ensemble of *N* atoms. Furthermore, the emitted photonic pulse peaks after a positive time delay^13,14^ that bears no direct dependence on the atom-field coupling strength, showing the collective interatomic motion to be a nonlinear process. Experimentally recorded on hydrofluoric gas^15^, cesium^16^, and most recently rubidium vapor^17^, this delay shows the necessity of a finite time during which the atoms establish cooperation before the radiation is initiated^18^.

Such a delay is also characteristic of synchronization: it takes finite time for the classical Huygens pendulums to reach in-phase oscillations. If one regards the correlated atoms as quantized Huygens oscillators concentrated on the lowest two levels at the weak-energy limit, it is not hard to find the resemblance between cooperated radiation and synchronization. In this paper, we formalize the study of this resemblance by analyzing the entanglement dynamics^19–21^ of two cavity-coupled qubits, which is the minimum-dimensional system that emulates the structure of an atomic ensemble undergoing cooperated radiation.

Many different metrics have been proposed over the years to measure synchronization of different quantum systems, such as a set of two-level systems in a shared bath^22^, quantum dots^23^, and resonator modes^24^. We note that synchronization as a quantum correlation can be studied using quantum discord^25^, as it was for two oscillators^26^, and non-locality. The advantage of entanglement approach is avoiding the unnecessary constraint to two-party correlation. We use specifically, in contrast to quantum discord, concurrence^27^ as the entanglement measure to quantify quantum synchronization in order to extract the temporal features of atomic cooperation. Concurrence is broadly applicable, especially for multipartite system. After it was introduced for two-qubit systems^27^, it was soon extended to the cases of three qubits^28^ and *N*-qubits^29^. Concurrence has then been generalized to two multi-level systems^30,31^ and finally to multipartite multi-level systems^32^. Given its generality, concurrence permits synchronization to be exemplified not only between two qubits, but among three parties that include the cavity field. In addition, synchronization is present in a Hilbert space of finite dimensions^33^, so non-locality is not an essential factor to initiate synchronization.

Further, concurrence is proved to be a good measure for static analysis of collective phenomena such as radiation^34,35^. Therefore, we extend the employment of multipartite concurrence to the dynamical analysis of the finite-dimensional cavity-qubit system. It is found that the entanglement among the components universally begins at a zero level and rises to a saturated value after a finite delay. The length of the delay and the saturated level depend nonlinearly on the cavity-qubit coupling strength. We use numerical simulations to show that the nonlinear dependence is divisible into weak, strong, and ultrastrong coupling regimes, verifying the role of operation regimes^36^ in the dynamics of superconducting circuits. Under all coupling strengths, the saturated entangled state is sustained thereafter, where oscillations in concurrence only exists locally about the saturation offset with an amplitude much smaller than the offset. These distinctive features of a synchronized state stand in constrast to the sudden death or death and revival^37^ one usually sees in entanglement evolution.

To verify the coincidence between the maximization of concurrence and the dynamic process of synchronization, we introduce a synchronization measure computed from the density matrix, which is modified from the synchronization measure introduced on the (*x*, *p*)-quadratures of quantum oscillators^24^. The transition point in time produced from the synchronization measures matches exactly the delay time found in the concurrence evolution, proving that the qubit-to-qubit synchronization is well registered in the entanglement. Moreover, since two-qubit systems on a superconducting circuit can produce superfluorescent pulses^38^, the synchronization delay is associated with the initiation of superradiance, thereby establishing the dynamic correlation between entanglement and the atomic cooperation for collective phenomena.

## Results

### The tripartite system

**Figure 1:**
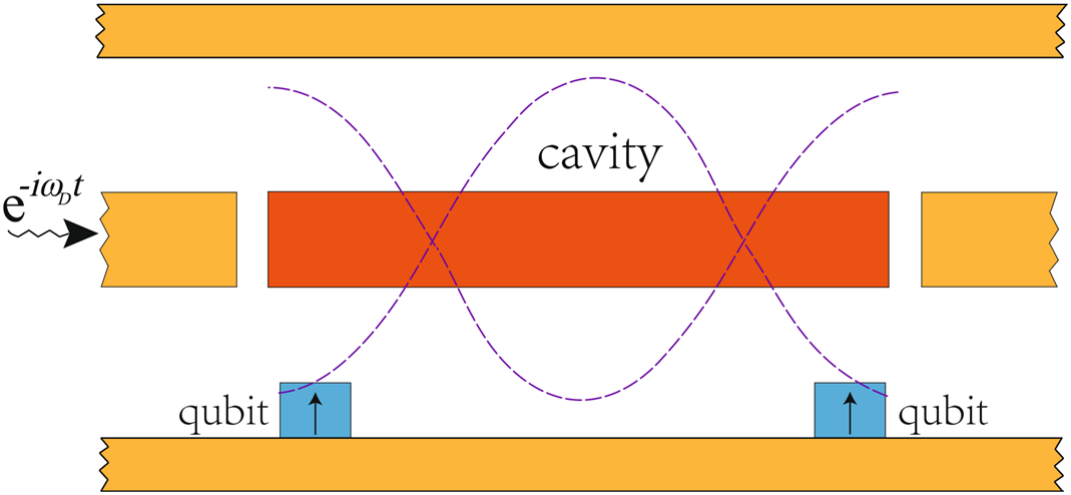
Illustration of the tripartite system: two superconducting qubits is coupled to the cavity field (indicated by the red strip), through which entanglement between the qubits are generated over time. The cavity field is driven by an incident field entered from the left.

The derivation for the dynamics discussed above is modeled on a generic cavity quantum electrodynamic (QED) system where each qubit is coupled to the cavity through dipole-field interaction under the rotating wave approximation. The parameters adopted for the numerical analysis are sourced from the superconducting circuit implementation^39,40^ of the cavity QED system. Hence, the tripartite system can be illustrated from the model Fig. 1, where the cavity is indicated by the stripped waveguide and the qubits are located at the anti-nodes of the cavity field to ensure maximum coupling.

The total system Hamiltonian $H=H_{0}+H_{\mathrm{int}}+H_{\mathrm {ext}}$ is composed of three parts: the free Hamiltonian, the interactions among the system components, and the external driving, which reads, respectively, $(\hbar =1)$

1 $$\begin{aligned} H_{0}&=\omega _{c}a^{\dagger }a+\Omega _{A}\sigma _{A,z} +\Omega _{B}\sigma _{B,z}, \end{aligned}$$

2 $$\begin{aligned} H_{\mathrm {int}}&=\eta _{A}\left( a\sigma _{A,+}+a^{\dagger }\sigma _{A,-}\right) +\eta _{B}\left( a\sigma _{B,+}+a^{\dagger }\sigma _{B,-}\right) , \end{aligned}$$

3 $$\begin{aligned} H_{\mathrm {ext}}&=i\varepsilon _{D}\left( a^{\dagger } e^{-i\omega _{D}t}-ae^{i\omega _{D}t}\right) . \end{aligned}$$

In $H_{0}$, $\omega _{c}$ denotes the frequency of the cavity mode and $\Omega _{A}$ ($\Omega _{B}$) denotes the transition frequency of the left (right) qubit, associated with the Pauli matrix $\sigma _{A,z}$ ($\sigma _{B,z}$). In $H_{\mathrm {int}}$, $\eta _{A}(\eta _{B})$ denotes the coupling strength to the left (right) qubit. In $H_{\mathrm {ext}}$, the external driving field has frequency $\omega _{D}$ and driving strength $\varepsilon _{D}$.

The combined system of a cavity and two qubits has its bare states described by the tensor product state $\{|e_{A}\rangle ,|g_{A}\rangle \}\otimes \{|n\rangle \}\otimes \{|e_{B}\rangle ,|g_{B}\rangle \}$, where $|e_{A}\rangle$ ($|g_{A}\rangle$) denotes the excited (ground) state of the left qubit; $|n\rangle$ denotes the Fock number states of the cavity mode; $|e_{B}\rangle$ ($|g_{B}\rangle$) denotes the excited (ground) state of the right qubit. To simplifying the notation, we omit the subscripts *A* and *B* when writing the product states and let the first letter denote the state of the left qubit, the middle letter that of the cavity mode, and the last letter that of the right qubit (e.g. $|e,n,g\rangle =|e_{A}\rangle \otimes |n\rangle \otimes |g_{B}\rangle$).

The free Hamiltonian $H_{0}$ and the interaction Hamiltonian $H_{\mathrm{int}}$ constitute a closed subsystem, for which there exist dressed states that diagonalize $H_{0}+H_{\mathrm {int}}$. To find an analytical expression for the dressed states, we consider the sets of energy-conserving states $|e,n,g\rangle$, $|g,n+1,g\rangle$, and $|g,n,e\rangle$, which are resonant within single-photon processes, to contribute to a dressed state for each *n*. In other words, the state $|e,n,e\rangle$ which is resonant with $|g,n+2,g\rangle$ through a double-photon process is omitted, allowing the derivation below to be purely analytical. Single-photon processes among the collective states of an ensemble of two-level systems are responsible for the majority of qubit operations useful for quantum information processing, which includes generating the entangled *W*-state^34^ and the multistable attractor states^41^. Such collective states are also experimentally proven on superconducting qubits^42^.

These single-photon resonant states form an invariant subspace, for which the closed Hamiltonian consists of $3\times 3$ symmetric block matrices. Therefore, we have the eigen-equation

4 $$\begin{aligned} \left( H_{\mathrm {0}}+H_{\mathrm{int}}\right) |u_{k}^{(n)}\rangle= & {} E_{k}^{(n)}\left| u_{k}^{(n)}\right\rangle , \end{aligned}$$

where the eigenvectors $|u_{k}^{(n)}\rangle$ denote the dressed states that diagonalize the $3n\times 3n$ matrix $H_{0}+H_{\mathrm {int}}$ and the eigenvalues $E_{k}^{(n)}$ denote the dressed-state energies in the diagonalized space. The index *k* enumerates $\{1,2,3\}$ to indicate the dressed levels within the *n*-th cluster.

Block-diagonalizing $H_{0}+H_{\mathrm {int}}$ for Eq. (4) results in a cubic equation of $E_{k}^{(n)}$ for each *n*, whose roots are

5 $$\begin{aligned} E_{k}^{(n)}=\frac{2}{3}\sqrt{\delta ^{2}+3(\Delta ^{2}+\eta _{A}^{2} +\eta _{B}^{2})}\cos \left( \theta +\frac{2k\pi }{3}\right) +n\omega _{c} +\frac{\delta }{3}. \end{aligned}$$

In the equation, $\Delta =\Omega _{A}-\Omega _{B}$ denotes the detuning between the two qubits and $\delta =\omega _{c}-\Omega _{A}-\Omega _{B}$ the detuning between the cavity and the two qubits combined. The angle $\theta$ is defined through its triple-angle formula

6 $$\begin{aligned} \cos (3\theta )=\frac{2\delta ^{3}+9\delta \left( \eta _{A}^{2} +\eta _{B}^{2}\right) -18\delta \Delta ^{2}+27\Delta \left( \eta _{A}^{2} -\eta _{B}^{2}\right) }{2\left[ \delta ^{2}+3(\Delta ^{2}+\eta _{A}^{2} +\eta _{B}^{2})\right] ^{3/2}}. \end{aligned}$$

The corresponding eigenvector reads

7 $$\begin{aligned} |u_{k}^{(n)}\rangle =\alpha _{A,k}^{(n)}|e,n,g\rangle +\alpha _{C,k}^{(n)} |g,n+1,g\rangle +\alpha _{B,k}^{(n)}|g,n,e\rangle , \end{aligned}$$

where the transformation coefficients are

8 $$\begin{aligned} \alpha _{A,k}^{(n)}&=-\eta _{A}(\Delta -n\omega _{c}+E_{k}^{(n)}) \Bigl /Z_{k}^{(n)}, \end{aligned}$$

9 $$\begin{aligned} \alpha _{C,k}^{(n)}&=\Bigl [\Delta ^{2}-(E_{k}^{(n)} -n\omega _{c})^{2}\Bigr ]\Bigl /Z_{k}^{(n)}, \end{aligned}$$

10 $$\begin{aligned} \alpha _{B,k}^{(n)}&=\eta _{B}(\Delta -E_{k}^{(n)}+n\omega _{c})\Bigl /Z_{k}^{(n)}, \end{aligned}$$

with $Z_{k}^{(n)}$ being the normalization constant

11 $$\begin{aligned} Z_{k}^{(n)}=\left( \eta _{A}^{2}\left[ \Delta +E_{k}^{(n)}- n\omega _{c}\right] ^{2}+\eta _{B}^{2}\Bigl [\Delta -E_{k}^{(n)} +n\omega _{c}\Bigr ]^{2}+\left[ \Delta ^{2}-(E_{k}^{(n)} -n\omega _{c})^{2}\right] ^{2}\right) ^{1/2}. \end{aligned}$$

The detailed derivation is given in the Methods section.

In the dressed space spanned by the basis vectors of Eq. (7), the closed Hamiltonian is written in the diagonalized form

12 $$\begin{aligned} H_{\mathrm {0}}+H_{\mathrm{int}}= & {} \sum _{n,k}E_{k}^{(n)}|u_{k}^{(n)}\rangle \langle u_{k}^{(n)}|, \end{aligned}$$

while the annihilation operator

13 $$\begin{aligned} a&={\mathbb {I}}_{A}\otimes a\otimes {\mathbb {I}}_{B}\nonumber \\&\approx \sum _{n}|g,n,e\rangle \langle g,n+1,e|+|e,n,g\rangle \langle e,n+1,g|+\sqrt{2}|g,n+1,g\rangle \langle g,n+2,g| \end{aligned}$$

under the single-photon processes is transformed to

14 $$\begin{aligned} a=\sum _{n,j,k}\Bigl [\alpha _{A,j}^{(n)*}\alpha _{A,k}^{(n+1)} +\sqrt{2}\alpha _{C,j}^{(n)*}\alpha _{C,k}^{(n+1)} +\alpha _{B,j}^{(n)*}\alpha _{B,k}^{(n+1)}\Bigl ]|u_{j}^{(n)}\rangle \langle u_{k}^{(n+1)}| \end{aligned}$$

where the indices *j* and *k* enumerate over the set $\{1,2,3\}$.

The permitted dressed level transitions induced by the external driving can be found by substituting Eq. (14) into Eq. (3). In the following, we consider the weak-energy limit where the transitions are confined to the lowest two clusters of states ($n=0$ and $n=1$). This consideration greatly simplify the numerical analysis of the evolution by confining the matrix representation of the equations of motion to 6 dimensions. It is also realistic since higher clusters require the much less likely occurrence of multi-photon resonance driven by the external $\varepsilon _{D}$. Though obtaining states of high photon number are possible with the assistance of auxiliary qubits^43^, they are much shorter-lived. We therefore write the total Hamiltonian as

15 $$\begin{aligned} H^{\left( 0,1\right) }&= \sum _{j}\Bigl [E_{j}^{(0)}|u_{j}^{(0)}\rangle \langle u_{j}^{(0)}|+E_{j}^{(1)}|u_{j}^{(1)}\rangle \langle u_{j}^{(1)}|\Bigr ]-\sum _{j,k}i\varepsilon _{D}e^{i\omega _{D}t}\nonumber \\&\quad \Bigl [\Bigl (\alpha _{A,j}^{(0)*}\alpha _{A,k}^{(1)} +\sqrt{2}\alpha _{C,j}^{(0)*}\alpha _{C,k}^{(1)} +\alpha _{B,j}^{(0)*}\alpha _{B,k}^{(1)}\Bigr )|u_{j}^{(0)}\rangle \langle u_{k}^{(1)}|+\mathrm {H.c.}\Bigl ] \end{aligned}$$

in the dressed space. Introducing the time-dependent state vector

16 $$\begin{aligned} \left| \psi (t)\right\rangle= & {} \sum _{j}\left( c_{j}(t)|u_{j}^{(0)}\rangle +d_{j}(t) |u_{j}^{(1)}\rangle \right) \end{aligned}$$

in the confined state space and applying it to the Hamiltonian above, one has the Schrödinger equations of the time coefficients

17 $$\begin{aligned} {\dot{c}}_{j}(t)= & {} -iE_{j}^{(0)}c_{j}(t)-\varepsilon _{D}e^{i\omega _{D}t}\lambda _{l} \alpha _{l,j}^{(0)*}\alpha _{l,k}^{(1)}d_{k}(t), \end{aligned}$$

18 $$\begin{aligned} {\dot{d}}_{j}(t)= & {} -iE_{j}^{(1)}d_{j}(t)+\varepsilon _{D}e^{-i\omega _{D}t}\lambda _{l} \alpha _{l,j}^{(1)*}\alpha _{l,k}^{(0)}c_{k}(t), \end{aligned}$$

where $\lambda _{l}$ denotes the weight of the summation over the index *l* for the system component *A* (the left qubit), *B* (the right qubit), or *C* (the cavity), i.e. $\lambda _{A}=\lambda _{B}=1$ and $\lambda _{C}=\sqrt{2}$. In the equations, we observe the Einstein summation convention.

In the rotating frame $c_{j}\left( t\right) =c_{j}^{\prime }\left( t\right) \mathrm {exp}\{-iE_{j}^{(0)}t\}$ and $d_{j}(t)=d_{j}^{\prime }(t)\mathrm {exp}\{-iE_{j}^{(1)}t\}$, the coupled equations can be written as the linear homogeneous system of differential equations $\dot{{\mathbf {c}}^{\prime }}=A{\mathbf {c}}^{\prime }$ where $\mathbf {c^{\prime }}=\left[ c_{1}^{\prime }\;c_{2}^{\prime } \;c_{3}^{\prime }\;d_{1}^{\prime }\;d_{2}^{\prime }\;d_{3}^{\prime }\right]$ and

19 $$  A = \left[ {\begin{array}{ll}    0 & { - \left[ {\varepsilon _{D} e^{{i\zeta _{{kj}} t}} \lambda _{l} \alpha _{{l,j}}^{{(0)*}} \alpha _{{l,k}}^{{(1)}} } \right]}  \\    {\varepsilon _{D} e^{{ - i\zeta _{{jk}} t}} \lambda _{l} \alpha _{{l,j}}^{{(1)*}} \alpha _{{l,k}}^{{(0)}} } & 0  \\   \end{array} } \right] $$

where the square brackets $[\cdot ]$ indicate $3\times 3$ submatrices in the matrix *A* with *j* and *k* being the row and the column indices, respectively. We denote $\zeta _{kj}=\omega _{D}-\left( E_{k}^{\left( 1\right) }-E_{j}^{\left( 0\right) }\right)$ for the detuning between the driving and the dressed states. Since *A* is integrable, then solving the linear system for $\{c_{j},d_{j}\}$ and expanding the dressed states by using the bare states in Eq. (16), one can find the expansion coefficients $\gamma$ of the state vector

20 $$\begin{aligned} |\psi (t)\rangle=& {} \gamma _{A}^{(0)}\left( t\right) |e,0,g\rangle +\gamma _{C}^{(0)}\left( t\right) |g,1,g\rangle +\gamma _{B}^{(0)} \left( t\right) |g,0,e\rangle \nonumber \\&+\,\gamma _{A}^{(1)}\left( t\right) |e,1,g\rangle +\gamma _{C}^{(1)} \left( t\right) |g,2,g\rangle +\gamma _{B}^{(1)} \left( t\right) |g,1,e\rangle \end{aligned}$$

back in the bare state space.

### Evolution of the state vector

To see that the evolution of the state vector can initiate the synchronization of the two delocalized qubits, we assume the cavity mode is initially driven by the external field to reach a partial population inversion while setting the qubits initially at the ground. In other words, the expansion coefficients at the initial moment are: $\gamma _{C}^{\left( 0\right) }=\sqrt{0.9}$, $\gamma _{C}^{\left( 1\right) }=\sqrt{0.1}$, and $\gamma _{A}^{\left( 0\right) }=\gamma _{B}^{\left( 0\right) }=\gamma _{A}^{\left( 1\right) }=\gamma _{B}^{\left( 1\right) }=0$.

**Figure 2:**
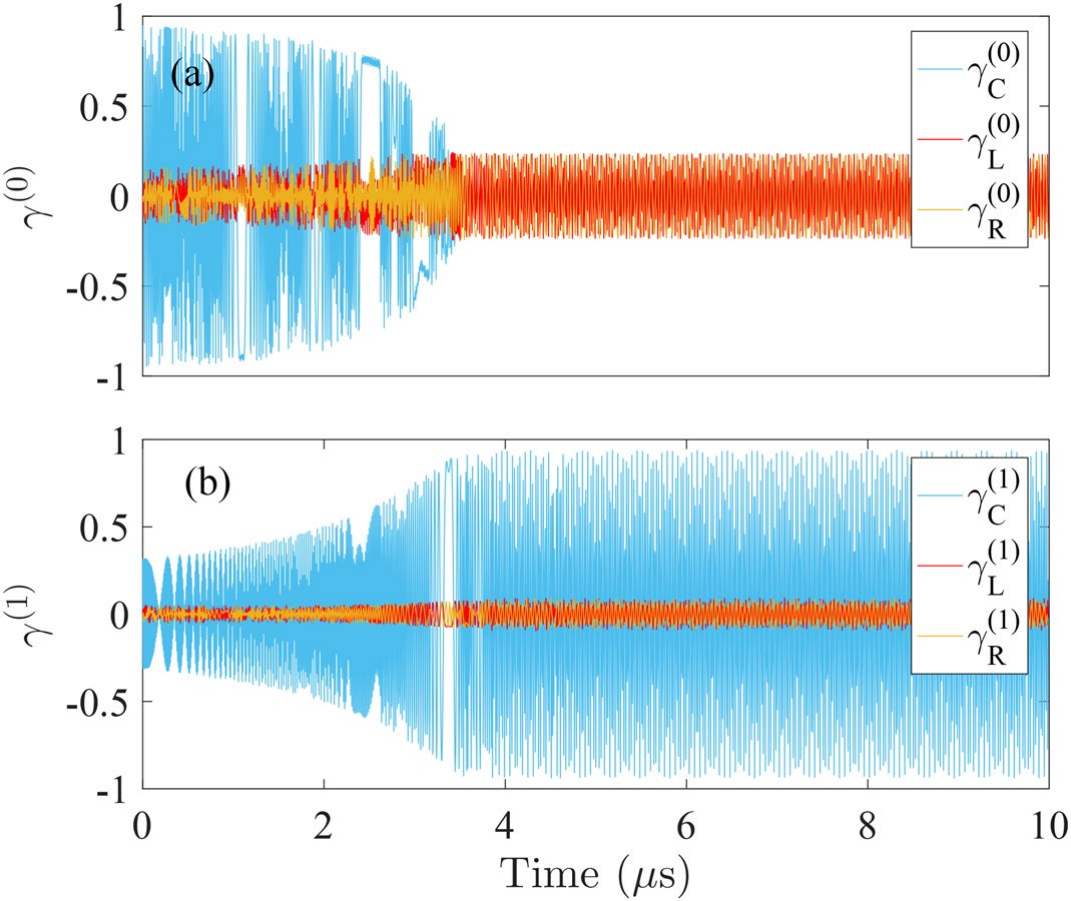
The time evolutions of the six expansion coefficients: (**a**) for $n=0$; (**b**) for $n=1$. The red, the blue, and the yellow curves associate with the state $|e,n,g\rangle$, $|g,n+1,g\rangle$, and $|g,n,e\rangle$, respectively.

We plot out the evolutions of these coefficients in Fig. 2, using the experimentally accessible parameters of superconducting charge-phase qubits^40^: $\Omega _{A}/2\pi =\Omega _{B}/2\pi =6.1$ GHz, $\eta _{A}/2\pi =\eta _{B}/2\pi =500$ MHz, and $\omega _{c}/2\pi =6.32$ GHz. The frequency of the external field is maintained at $\omega _{D}/2\pi =5.3$ GHz. The lower set of states with $n=0$ is given in Fig. 2a whereas the upper set with $n=1$ is given in Fig. 2b. Note that, for the implementation of cavity QED system on a superconducting circuit, the cavity field refers to the standing microwave field that is sustained in the stripline (Cf. Fig. 1) and coupled capacitively to the Josephson-junction qubit. Hence, by distancing qubits and placing them at different positions relative to the cavity nodes, it is possible to realize arbitrary cavity-qubit interactions. For instance, maximal coupling can be obtained by placing the qubits at antinodes^42^.

We observe that for both the lower set and the upper set of states, there exists a transition point of the oscillations of the coefficients, which is located at about $3.7\mu \mathrm {s}$ in the plots. In particular, $\gamma _{C}^{\left( 0\right) }$ is transited from a region of shrinking oscillation to a region of small fluctuation at this point. Meanwhile, $\gamma _{A}^{\left( 1\right) }$,$\gamma _{B}^{\left( 1\right) }$, and $\gamma _{C}^{\left( 1\right) }$ are transited from an amplifying region to a region of saturated oscillation envelope. The contrasting behavior of the two sets of coefficients demonstrates that the energy excitation that exists in the cavity mode is transferred to the left and the right qubits whose complementary oscillations imply the build-up of the entanglement between them.

### Bipartite and tripartite concurrences

To fully capture the evolution characteristics of the two cavity-coupled qubits from a holistic point of view, we apply two entanglement measures—bipartite concurrence and tripartite concurrence—to the state vector of the total system.

The bipartite concurrence quantifies the inseparability of the joint pure state of two coupled systems of arbitrary dimensions by inverting the density matrix. For our case here, the joint state is the product state $|\psi _{AB}\rangle$ of the indirectly coupled left and right qubits. Thus the inversion is conducted through the superoperator $S_{D_{1}}\otimes S_{D_{2}}$ where the dimensions $D_{1}=D_{2}=2$ and the bipartite concurrence is defined as $C_{2}(\psi _{AB})=\sqrt{\langle \psi _{AB}|S_{2}\otimes S_{2}\left( |\psi _{AB}\rangle \langle \psi _{AB}|\right) |\psi _{AB}\rangle }$. Given the consideration of pure states, for which $\mathrm {tr}\rho ^{2}=1$ and $\mathrm {tr}\rho _{A}^{2}=\mathrm {tr}\rho _{B}^{2}$, the definition reduces to $C_{2}(\psi _{AB})=\sqrt{2\left[ 1-\mathrm {tr}\left( \rho _{A}^{2}\right) \right] }$ where $\rho _{A}=\mathrm {tr}_{B}\left( \mathrm {tr}_{C}\left( |\psi \rangle \langle \psi |\right) \right)$ is the reduced density matrix of the left qubit.

**Figure 3:**
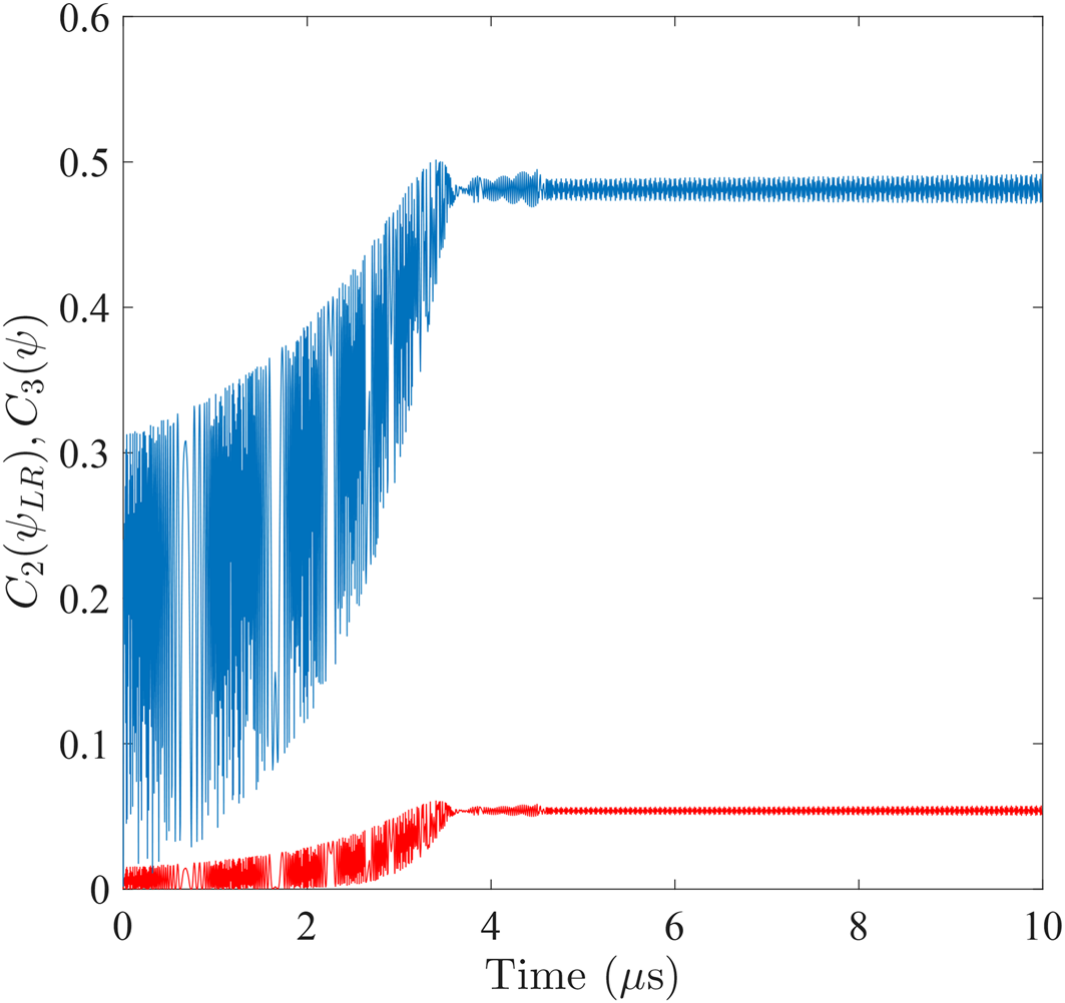
Time evolution of the bipartite concurrence $C_{2}\left( \psi _{AB}\right)$ between the two qubits (blue) and the tripartite concurrence $C_{3}\left( \psi \right)$ among the qubits and the cavity (red). A symmetric scenario is assumed between the left and the right qubits: $\eta _{A}/2\pi =\eta _{B}/2\pi =500$ MHz and $\Omega _{A}/2\pi =\Omega _{B}/2\pi =6.1$ GHz.

Applying $|\psi \left( t\right) \rangle$ in Eq. (20) to the formula, we derive the evolution of the bipartite concurrence, shown as the blue curve in Fig. 3. It becomes apparent that the transition point that manifests in Fig. 2a,b signifies the concurrence reaching a maximum after a gradual monotonic increase in the oscillation envelope. This maximum concurrence is retained thereafter. The finite delay time $\tau _{D}=3.7\mu \mathrm {s}$ that the concurrence spends to reach its maximal value reflects the time the two qubits use to reach a maximal synchronization through their mutual couplings to the cavity mode.

The cavity mode plays an active part in initiating the entanglement between the two qubits. From the entanglement-theoretic point of view, the concurrence is distributed among the qubits as well as the cavity. Taking away the pairwise entanglements between any two parties in the tripartite system, one obtains the residual concurrence that remains as an equally distributed entanglement among all three parties^28^. Extending the original formulation on three-qubit systems, we generalize the inversion operations for two arbitrary-dimensional systems given above to three arbitrary-dimensional system. That is, we introduce the superoperator

21 $$\begin{aligned} S_{D_{1}}\otimes S_{D_{2}}\otimes S_{D_{3}}\left( \rho \right)&= I\otimes I\otimes I-I\otimes I\otimes \rho _{B}-\rho _{A}\otimes I\otimes I-I\otimes \rho _{C}\otimes I\nonumber \\&\quad +\,\rho _{AB}\otimes I+I\otimes \rho _{CB}+\rho _{AC}\otimes I-\rho . \end{aligned}$$

for our $D_{1}\times D_{2}\times D_{3}$ dimension tripartite system, where $D_{1}=D_{3}=2$ for the qubits and $D_{2}=n$ for the cavity mode. In Eq. (21), *I* denotes the identity matrix while $\rho _{A}$, $\rho _{C}$, $\rho _{B}$, $\rho _{AB}$, $\rho _{CB}$, and $\rho _{AC}$ denote the reduced density matrices of the components and the two-component subsystems. Applying the inversion, we thus derive a tripartite residual concurrence

22 $$\begin{aligned} C_{3}\left( \psi \right)&=\sqrt{\langle \psi |S_{D_{1}}\otimes S_{D_{2}}\otimes S_{D_{3}}\left( |\psi \rangle \langle \psi |\right) |\psi \rangle }\nonumber \\&=\sqrt{1-\mathrm {tr}\rho _{B}^{2}-\mathrm {tr}\rho _{A}^{2}-\mathrm {tr} \rho _{C}^{2}+\mathrm {tr}\rho _{AB}^{2}+\mathrm {tr}\rho _{CB}^{2} +\mathrm {tr}\rho _{AC}^{2}-\mathrm {tr}\rho ^{2}}. \end{aligned}$$

Again, using Eq. (20), we plot the tripartite concurrence as the red curve in Fig. 3. One can verify from the plot that the residual concurrence evolves in a similar fashion, which contains a signifying transition point at the exactly same location $\tau _{D}$ as that of the bipartite concurrence. Before $\tau _{D}$, it arises from a zero value under a similarly increasing envelope whereas, after $\tau _{D}$, it retains a non-zero saturated value. The identical delay time again demonstrates the duration that the system components spend on cooperation before maximal synchronization is reached.

Comparing Figs. 2 and 3, one sees that the energy quantum first dwells on the cavity mode ($|g,1,g\rangle$ and $|g,2,g\rangle$) without being emitted and absorbed by the qubits. Only when the two qubits start to establish a cooperated motion does the qubit-cavity-qubit resonance become effective such that the qubits be excited to their respective excited states $|e,1,g\rangle$ and $|g,1,e\rangle$. The entanglement is also established among the three components when the excitation commences.

**Figure 4:**
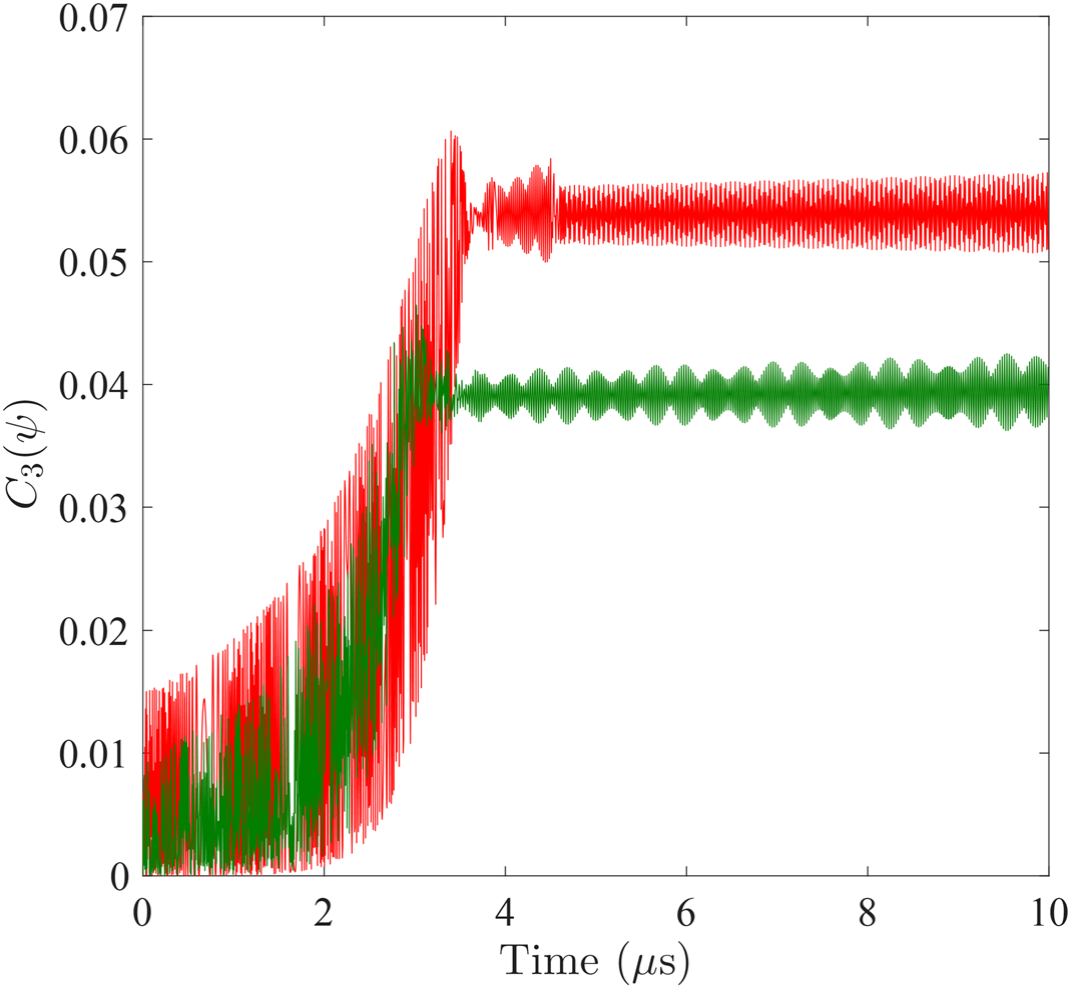
Comparison of the time evolutions of the tripartite concurrences between the symmetric (red curve) and the asymmetric (green curve) scenarios. For the symmetric case, both qubits are set to $\Omega _{A}/2\pi =\Omega _{B}/2\pi =6.1~\mathrm {GHz}$. For the asymmetric case, the right qubit is adjusted to $\Omega _{B}/2\pi =7.1$GHz. Coupling strengths are retained at $\eta _{A}/2\pi =\eta _{B}/2\pi =500$MHz throughout.

The concurrences plotted in Fig. 3 are computed upon a symmetric setting of system parameters: the level spacings and the coupling strengths of the qubits are assumed identical. The tripartite concurrence of an asymmetric scenario with the right qubit level spacing raised to $\Omega _{B}/2\pi =7.1\,\mathrm {GHz}$ is shown as the green curve in Fig. 4 while the rest of the parameters remain unchanged. For comparison, the symmetric case is plotted as the red curve in the background. We observe that the delay to saturated synchronization is inversely correlated with the larger eigenfrequency out of the two qubits. For the case in Fig. 4, increasing $\Omega _{B}$ reduces delay time $\tau _{D}$. On the other hand, symmetric settings lead to maximal synchronization at saturation. The asymmetric case given by the green curve has the saturated synchronization reduced to a lower level. Simulation under parameters set to varied values (not shown in figures) verify these observations.

We note that the dynamics originated from the equations of motion (17)–(18) does not take into consideration the relaxation effects which would require a master equation approach under the presence of an environment. Their omission greatly simplifies our derivations and assists the illustration of the temporal features during the synchronization. These features would not be obscured even when environmental effects are observed since their time scales are much shorter than accessible coherence times on superconducting circuits. For instance, the synchronization delays in both settings of Fig. (4) appear within the first 4 µs after initiation. Whereas, superconducting qubits can achieve coherence time up to 100 µs using fabrication techniques^44^ and even up to hundreds of µs when an error-correcting feedback mechanism is implemented^45^.

**Figure 5:**
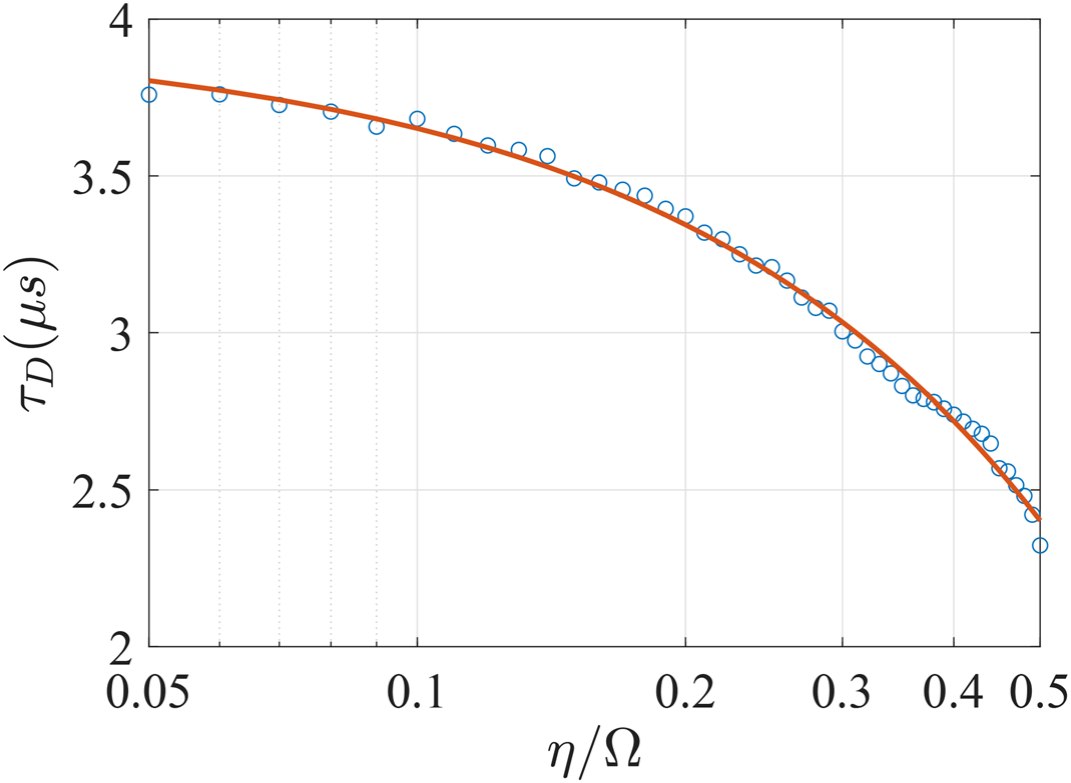
The time delay $\tau _{D}$ is plotted as a function of the dimensionless coupling strength $\eta /\Omega$ in a semilog scale. Symmetric setting is assumed: the qubit level spacings $\Omega /2\pi =\Omega _{A,B}/2\pi =6.1$ GHz and the coupling strength $\eta =\eta _{A,B}$. The circles indicate the data points in the simulation runnings.

The synchronization between the qubits is also affected by how strong they are driven by the cavity mode, i.e. the coupling strengths $\eta _{A}$ and $\eta _{B}$. Shown in Fig. 5 for the symmetric scenario $\eta _{A}=\eta _{B}$ in a semilog plot, the greater is the coupling, the lesser is the delay $\tau _{D}$. The numerical fit shows that the delay obeys a quadratic relation over the exponential increase in coupling strength. After the delay, a shorter delay time is associated with a higher saturated level of concurrence, showing a stronger synchronization between the qubits are reflected in both short delay and higher entanglement measure.

### Asynchronicity

Multi-partite concurrence as a measure of synchronization reveals a gradual increase between two qubits in the time domain, explaining the existence of a delay in the superfluorescent pulse of cooperated radiation from a system-intrinsic point of view. This synchronization is affected by many factors, among which the symmetry of the system parameters plays an important part. Tuning the system from a symmetric setting to an asymmetric setting is accompanied by tuning the transition rates of the qubits from a synchronous setting to an asynchronous setting. For the latter, we refer to the scenario where the population of the left qubit oscillates at a Rabi frequency not synchronous to that of the right qubit.

However, since two oscillators sharing a common oscillating platform are able to synchronize after certain time duration according to classical mechanics, we expect the qubits sharing the cavity resonator would behave similarly. To precisely describe the transition process from asynchronous regime to synchronous regime, we extend the quantum synchronization measure introduced in Ref.^24^ for continuous variable systems to discrete systems. We consider instead the measure of asynchronicity

23 $$\begin{aligned} {\mathcal {A}}\left( t\right) =\Bigl |\det (\rho _{A}(t)-\rho _{B}(t))\Bigr |. \end{aligned}$$

that compares the difference between two density matrices for two two-level systems.

**Figure 6:**
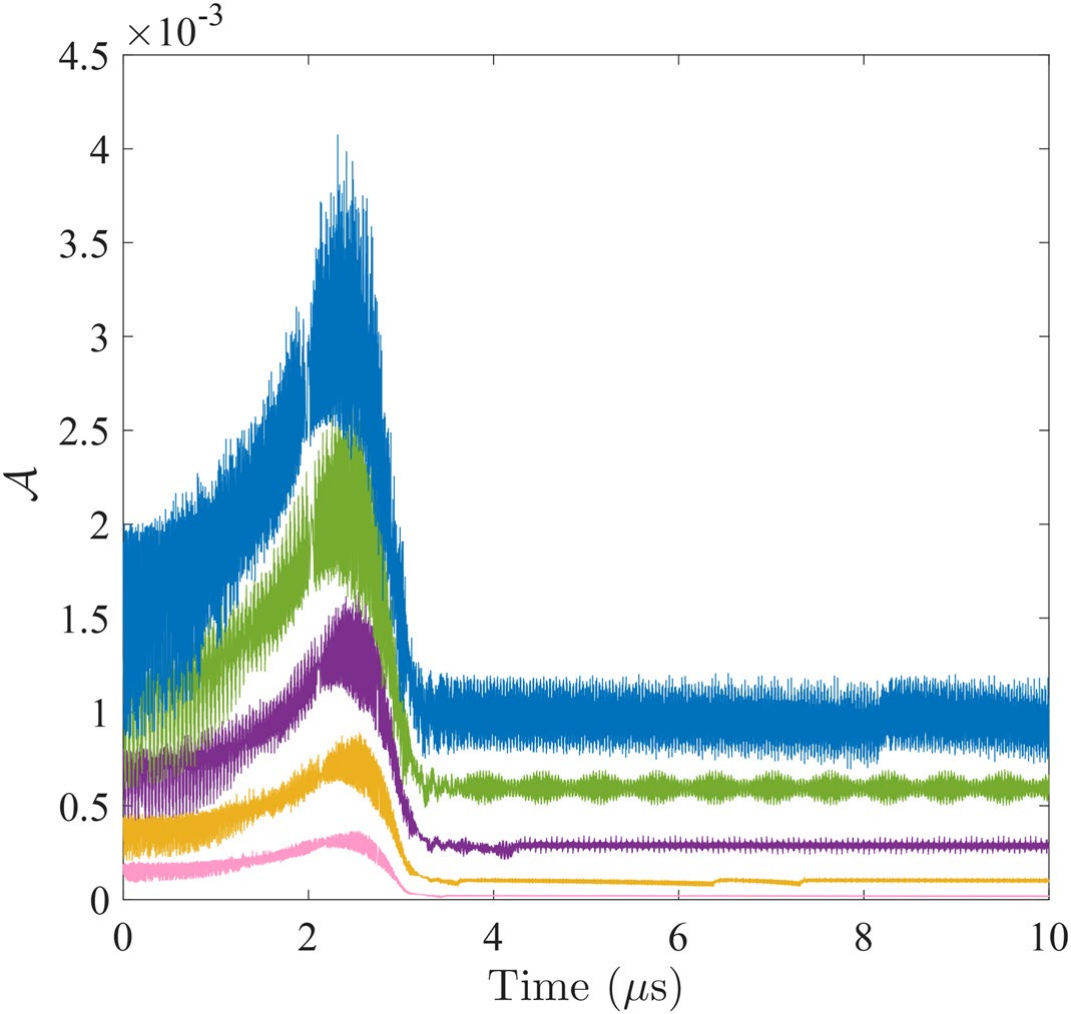
Asynchronization ${\mathcal {A}}$ of two cavity-coupled qubits under five different coupling strengths: $\eta _{A}/2\pi =\eta _{B}/2\pi =200$MHz (pink), 300MHz (yellow), 400MHz (purple), 500MHz (green), and 600MHz (blue). The qubit level spacings are kept at the asymmetric setting $\Omega _{A}/2\pi =6.1~\mathrm {GHz}$ and $\Omega _{B}/2\pi =7.1~\mathrm {GHz}$.

To consider the scenario where the sychronization is initiated from the cavity field as the medium, we let the initial state be only populated in the first and second excited state of the cavity mode, i.e. $|\psi (0)\rangle =3|g,1,g\rangle /\sqrt{10}+|g,2,g\rangle /\sqrt{10}$. In a superconducting circuit where the cavity mode corresponds to the photonic number state of the stripline resonator, the population is controlled through a resonant microwave pulse, which has a time duration commensurate with the cavity Rabi period, to be fed into the waveguide input^46^. For example, a $\pi$-pulse sends half of the population from $\left| 0\right\rangle$ to $\left| 1\right\rangle$. If imaginary phases in the coefficients of the eigenstates are desired, they can be fine-tuned with the assistance of an auxiliary qubit^43^. $\left| 2\right\rangle$ is here partially populated to emulate the resonance process where the equally spaced levels of the cavity mode has $\left| 1\right\rangle$ resonant with $\left| 2\right\rangle$ simultaneously with $\left| 0\right\rangle$. Given the initial condition, we find that a symmetric setting $\Omega _{A}=\Omega _{B}$ would always lead to a zero asynchronicity throughout independent of the coupling strengths $\eta _{A}$ and $\eta _{B}$. When $\Omega _{A}\ne \Omega _{B}$, the asymmetry leads to coupling-dependent asynchronicity, as shown by the plots given in Fig. 6 for five settings of coupling strengths.

No matter the coupling strength, there exists a transition point after which the asynchronicity remains at a stable value. This transition point is identical to the transition point shown in Fig. 4 (green curves) where the tripartite concurrence reaches a maximal value. The coincidence verifies our expectation that the synchronization is maximized when the asynchronicity is minimized. Therefore, synchronization between two qubits reflects the dynamic identity of the two qubits.

**Figure 7:**
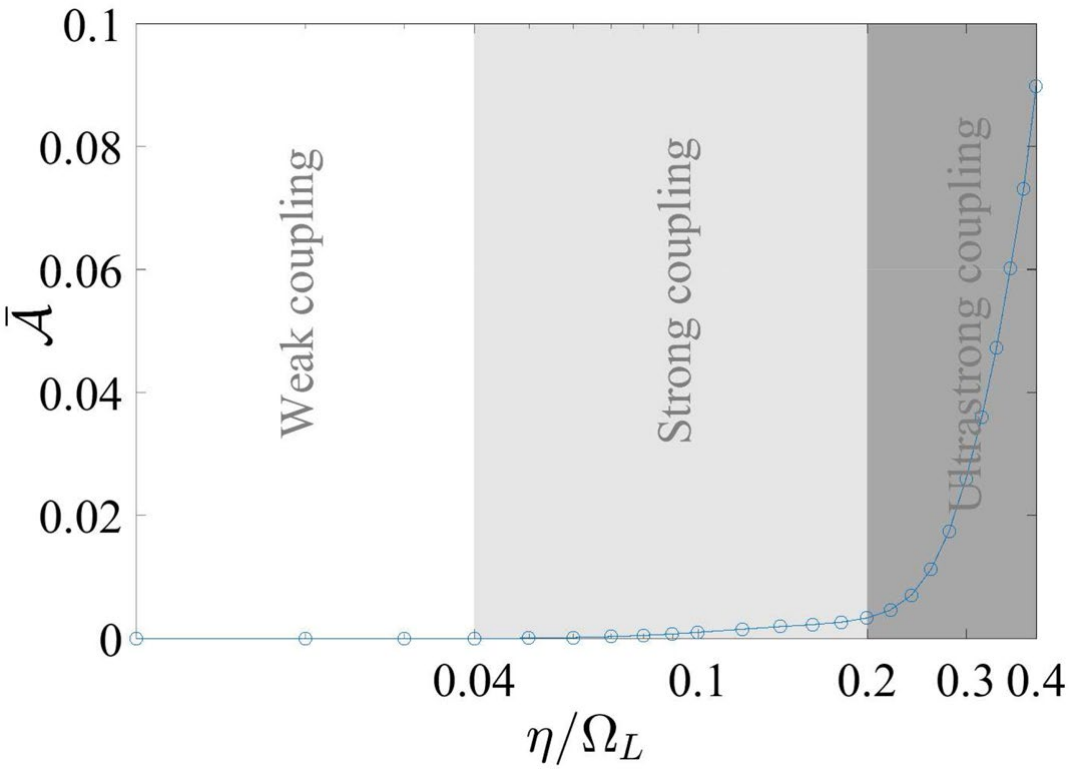
The stable value $\bar{{\mathcal {A}}}$ is plotted as a function of the coupling strength $\eta =\eta _{A}=\eta _{B}$ in the logarithmic dimensionless scale of $\eta /\Omega _{A}$, where the qubit level spacings are kept at the asymmetric setting $\Omega _{A}/2\pi =6.1~\mathrm {GHz}$ and $\Omega _{B}/2\pi =7.1~\mathrm {GHz}$. The circles indicate the data points from the simulation runnings.

Before reaching the stable minimal value, the asynchronicity increases from a non-zero value for a certain duration, which are spent on the cooperation by the qubits. When the coupling is sufficiently weak (below $\eta \approx 200$ MHz), the minimal stable value is almost vanishing (below $10^{-5}$). When the coupling becomes stronger, the feedback from the cavity mode to each of the qubits becomes adverse to the synchronizing motion. However, the feedback effect is not linear. Plotted as a function of the dimensionless coupling strength $\eta /\Omega _{A}$ at $\Omega _{A}/2\pi =6.1~\mathrm {GHz}$ and $\Omega _{B}/2\pi =7.1~\mathrm {GHz}$ in Fig. 7, the stable minimal value $\bar{{\mathcal {A}}}$ of asynchronicity first retains a negligible value in the weak coupling regime. At about $\eta /\Omega _{A}=0.04$, ${\bar{\mathcal {A}}}$ starts to increase slowly until it reaches a turning point at $\eta /\Omega _{A}=0.2$. The region between $\eta /\Omega _{A}=0.04$ and $\eta /\Omega _{A}=0.2$ can be regarded as a strong coupling regime for synchronization. After that, $\bar{{\mathcal {A}}}$ enters the ultra-strong coupling regime and increases with the coupling again until $\eta /\Omega _{A}\approx 0.33$, where stable minimal value of ${\mathcal {A}}$ is no longer discernible.

## Discussion

In conclusion, we have studied the synchronization between the two cavity-coupled qubits using multiple concurrence measures and asynchronicity. These real-valued measures are computed as functionals of dressed state vectors that evolve in time as they are driven by an external field. In all these measures acting as time functions, we obtain consistent features of transitions from an arbitrary initial system state to a final synchronized state. The transition in time reveals a synchronization delay that the qubits use to initiate superfluorescent pulse radiation, which explains the cooperation origin of the collective effect of superradiance.

The characteristics of the synchronization process, including the delay and the value of the stabilized asynchronicity, are highly dependent on the coupling strengths of the qubits relative to the their level spacings. They demonstrate from the entanglement perspective the different behaviors that the circuit QED systems adopt when operating in weak, strong, and ultra-strong coupling regimes. In general, synchronization occurs only in the strong- and ultrastrong-coupling regimes whereas its level of synchronization at the final state increases exponentially with the qubit-cavity coupling strength.

## Methods

For each $3\times 3$ block submatrix of the LHS of Eq. (4), we first split it into the sum of two matrices, one being the identity matrix $n\omega _{c}I$. The other matrix is the one to be diagonalized, the determinant equation of which is

24 $$\begin{aligned} \begin{vmatrix}\begin{array}{ccc} \Delta -\lambda &{} \eta _{A} &{} 0\\ \eta _{A} &{} \delta -\lambda &{} \eta _{B}\\ 0 &{} \eta _{B} &{} -\Delta -\lambda \end{array}\end{vmatrix}=0 \end{aligned}$$

where $\lambda$ denotes the eigenvalue to be determined, i.e. $E_{k}^{(n)}=\lambda +n\omega _{c}$.

This determinant equation is equivalent to the cubic equation

25 $$\begin{aligned} \lambda ^{3}-\delta \lambda ^{2}-\left( \Delta ^{2}+\eta _{A}^{2} +\eta _{B}^{2}\right) \lambda +\delta \Delta ^{2} -\Delta \left( \eta _{A}^{2}-\eta _{B}^{2}\right) =0, \end{aligned}$$

whose roots can be derived by absorbing the quadratic term through the transform $\lambda =x+\delta /3$. The transformed equation becomes $x^{3}+px+q=0$, where

26 $$\begin{aligned} p&=-\left( \frac{1}{3}\delta ^{2}+\Delta ^{2}+\eta _{A}^{2}+\eta _{B}^{2}\right) ,\ \end{aligned}$$

27 $$\begin{aligned} q&=-\frac{2}{27}\delta ^{3}-\frac{1}{3}\delta \left( \eta _{A}^{2} +\eta _{B}^{2}\right) +\frac{2}{3}\delta \Delta ^{2}-\Delta \left( \eta _{A}^{2}-\eta _{B}^{2}\right) . \end{aligned}$$

In the close cavity-qubit resonance region $\delta \thickapprox 0$, the discriminant *D* is simplified to

28 $$\begin{aligned} D= & {} \frac{1}{4}\Delta ^{2}\left( \eta _{A}-\eta _{B}\right) ^{4} -\frac{1}{27}\left( \Delta ^{2}+\eta _{A}^{2}+\eta _{B}^{2}\right) ^{3}. \end{aligned}$$

To let the cubic equation admit three non-degenerate real roots, we consider the range

29 $$\begin{aligned} \left( 3-2\sqrt{2}\right) \eta _{B}<&\eta _{A}&<\left( 3+2\sqrt{2}\right) \eta _{B} \end{aligned}$$

that makes $D<0$. Applying the Vieta’s formula, the roots *x* can be found with a parametric angle $\theta$ given by Eq. (6).

Using the eigenvalues given in Eq. (5) and expanding the eigenvector $\left| u_{k}^{(n)}\right\rangle$ in the bare state space given by Eq. (7), we have the column matrix equation

30 $$\begin{aligned} \left[ \begin{array}{ccc} \Delta -\lambda &{} \eta _{A} &{} 0\\ \eta _{A} &{} \delta -\lambda &{} \eta _{B}\\ 0 &{} \eta _{B} &{} -\Delta -\lambda \end{array}\right] \left[ \begin{array}{c} \alpha _{A}\\ \alpha _{C}\\ \alpha _{B} \end{array}\right] =0. \end{aligned}$$

Letting $\alpha _{C}$ be the proportional constant, we find $\alpha _{A}=-\eta _{A}\alpha _{C}\Bigl /(\Delta -\lambda )$ and $\alpha _{B}=\eta _{B}\alpha _{C}\Bigl /(\Delta +\lambda )$. Then normalizing the coefficients, their expressions are given by Eqs. (8)–(10) for the *k*-th energy level within the *n*-th cluster.

## References

[1] Pikovsky A, Kurths J, Rosenblum M, Kurths J (2003). Synchronization: A Universal Concept in Nonlinear Sciences.

[2] Acebron JA, Bonilla LL, Perez Vicente CJ, Ritort F, Spigler R (2005). The Kuramoto model: a simple paradigm for synchronization phenomena. Rev. Mod. Phys..

[3] Shim S-B, Imboden M, Mohanty P (2007). Synchronized oscillation in coupled nanomechanical oscillators. Science.

[4] Vinokur VM (2008). Superinsulator and quantum synchronization. Nature.

[5] Zhirov OV, Shepelyansky DL (2008). Synchronization and bistability of a qubit coupled to a driven dissipative oscillator. Phys. Rev. Lett..

[6] Ying L, Lai Y-C, Grebogi C (2014). Quantum manifestation of a synchronization transition in optomechanical systems. Phys. Rev. A.

[7] Qiao G, Gao H, Liu H, Yi XX (2018). Quantum synchronization of two mechanical oscillators in coupled optomechanical systems with Kerr nonlinearity. Sci. Rep..

[8] Heimonen H, Kwek LC, Kaiser R, Labeyrie G (2018). Synchronization of a self-sustained cold-atom oscillator. Phys. Rev. A.

[9] Walter S, Nunnenkamp A, Bruder C (2014). Quantum synchronization of a driven self-sustained oscillator. Phys. Rev. Lett..

[10] Bonifacio R, Lugiato LA (1975). Phys. Rev. A.

[11] Bonifacio R, Lugiato LA (1978). ibid.

[12] Dicke RH (1954). Coherence in spontaneous radiation processes. Phys. Rev..

[13] Haake F (1979). Macroscopic quantum fluctuations in superfluorescence. Phys. Rev. Lett..

[14] Polder D, Schuurmans MFH, Vrehen QHF (1979). Superfluorescence: quantum-mechanical derivation of Maxwell–Bloch description with fluctuating field source. Phys. Rev. A.

[15] Skribanowitz N, Herman IP, MacGillivray JC, Feld MS (1973). Observation of Dicke superradiance in optically pumped HF gas. Phys. Rev. Lett..

[16] Gibbs HM, Vrehen QHF, Hikspoors HMJ (1977). Single-pulse superfluorescence in cesium. Phys. Rev. Lett..

[17] Ariunbold GO (2010). Observation of picosecond superfluorescent pulses in rubidium atomic vapor pumped by 100-fs laser pulses. Phys. Rev. A.

[18] Arecchi FT, Courtens E (1970). Cooperative phenomena in resonant electromagnetic propagation. Phys. Rev. A.

[19] Abdi M, Pirandola S, Tombesi P, Vitali D (2012). Entanglement swapping with local certification: application to remote micromechanical resonators. Phys. Rev. Lett..

[20] Tian L (2013). Robust photon entanglement via quantum interference in optomechanical interfaces. Phys. Rev. Lett..

[21] Huan T, Zhou R, Ian H (2015). Dynamic entanglement transfer in a double-cavity optomechanical system. Phys. Rev. A.

[22] Yokoshi N, Odagiri K, Ishikawa A, Ishihara H (2017). Synchronization dynamics in a designed open system. Phys. Rev. Lett..

[23] Greilich A (2006). Mode locking of electron spin coherences in singly charged quantum dots. Science.

[24] Mari A, Farace A, Didier N, Giovannetti V, Fazio R (2013). Measures of quantum synchronization in continuous variable systems. Phys. Rev. Lett..

[25] Ollivier H, Zurek WH (2001). Quantum discord: a measure of the quantumness of correlations. Phys. Rev. Lett..

[26] Giorgi GL, Galve F, Manzano G, Colet P, Zambrini R (2012). Quantum correlations and mutual synchronization. Phys. Rev. A.

[27] Wootters WK (1998). Entanglement of formation of an arbitrary state of two qubits. Phys. Rev. Lett..

[28] Coffman V, Kundu J, Wootters WK (2000). Phys. Rev. A.

[29] Hashemi Rafsanjani SM, Huber M, Broadbent CJ, Eberly JH (2012). Genuinely multipartite concurrence of $$N$$-qubit $$X$$-matrices. Phys. Rev. A.

[30] Rungta P, Buzek V, Caves CM, Hillery M, Milburn GJ (2001). Universal state inversion and concurrence in arbitrary dimensions. Phys. Rev. A.

[31] Mintert F, Kuś M, Buchleitner A (2004). Concurrence of mixed bipartite quantum states in arbitrary dimensions. Phys. Rev. Lett..

[32] Mintert F, Kuś M, Buchleitner A (2005). Concurrence of mixed multipartite quantum states. Phys. Rev. Lett..

[33] Roulet A, Bruder C (2018). Synchronizing the smallest possible system. Phys. Rev. Lett..

[34] Ian H (2016). Quasi-lattices of qubits for generating inequivalent multipartite entanglements. Europhys. Lett..

[35] Ian H, Liu Y (2014). Cavity polariton in a quasilattice of qubits and its selective radiation. Phys. Rev. A.

[36] Forn-Díaz P (2017). Ultrastrong coupling of a single artificial atom to an electromagnetic continuum in the nonperturbative regime. Nat. Phys..

[37] Yu T, Eberly JH (2004). Finite-time disentanglement via spontaneous emission. Phys. Rev. Lett..

[38] Mlynek JA, Abdumalikov AA, Eichler C, Wallraff A (2014). Observation of Dicke superradiance for two artificial atoms in a cavity with high decay rate. Nat. Commun..

[39] Blais A, Huang R-S, Wallraff A, Girvin SM, Schoelkopf RJ (2004). Cavity quantum electrodynamics for superconducting electrical circuits: an architecture for quantum computation. Phys. Rev. A.

[40] Majer J (2007). Coupling superconducting qubits via a cavity bus. Nature.

[41] Ian H (2017). Stability branching induced by collective atomic recoil in an optomechanical ring cavity. New J. Phys..

[42] Fink JM (2009). Dressed collective qubit states and the Tavis–Cummings model in circuit QED. Phys. Rev. Lett..

[43] Hofheinz M (2009). Synthesizing arbitrary quantum states in a superconducting resonator. Nature.

[44] Rigetti C (2012). Superconducting qubit in a waveguide cavity with a coherence time approaching 0.1 ms. Phys. Rev. B.

[45] Shankar S (2013). Autonomously stabilized entanglement between two superconducting quantum bits. Nature.

[46] Niskanen AO (2007). Quantum coherent tunable coupling of superconducting qubits. Science.

